# Discrimination of Traditional Chinese Medicine Syndromes in Type 2 Diabetic Patients Based on Metabolomics-Proteomics Profiles

**DOI:** 10.1155/2023/5722131

**Published:** 2023-06-02

**Authors:** Xin Shao, Gang Hu, Yuanyuan Lu, Ming Li, Baohua Shen, Wenwen Kong, Yanhua Guan, Xin Yang, Jia Fang, Jing Liu, Yingzhuo Ran

**Affiliations:** Department of Endocrinology, Nanjing Hospital of Traditional Chinese Medicine, Nanjing 210012, China

## Abstract

**Materials and Methods:**

The metabolomics-proteomics of sixty patients with T2DM were acquired by high-performance liquid chromatography (HPLC). In addition, some clinical features, containing total cholesterol (TC), triglycerides (TG), hemoglobin A1c (HbA1c), body mass index (BMI), and low-density lipoprotein (LDL) together with high-density lipoprotein (HDL), were determined via clinical detection strategies. Abundant metabolites and proteins, respectively, were identified with the analysis of liquid chromatography tandem mass spectrometry (LC-MS/MS).

**Results:**

22 differentially abundant metabolites and 15 differentially abundant proteins were determined. The analysis of bioinformatics suggested that the differentially abundant proteins were commonly associated with the renin-angiotensin system, vitamin digestion and absorption, hypertrophic cardiomyopathy, and so on. Furthermore, differentially abundant metabolites were amino acids and were associated with the biosynthesis of CoA and pantothenate, together with the metabolisms of phenylalanine, beta-alanine, proline, and arginine. Combination analysis revealed that the vitamin metabolism pathway was predominantly affected.

**Conclusions:**

DHS syndrome can be separated by certain metabolic-proteomic differences, and metabolism is particularly prominent, especially in vitamin digestion and absorption. From the molecular level, we provide preliminary data for the extensive application of TCM in the study of T2DM, and at the same time benefited in a sense diagnosis and treatment of T2DM.

## 1. Introduction

Diabetes is a serious health problem in the world, and the management costs of the national health system and patients are high. The latent effect of diabetes on health care systems, health, and life expectancy along with financial costs will increase over the next few years [1]. The pathogenesis of T2DM is not entirely clear. It is the most common type of diabetes characterized through impaired *β*-cell function, insulin resistance (IR), chronic hyperglycemia, as well as comorbidities for instance cardiovascular disease and obesity [2]. Treatment of T2DM needs the sequence of latent measures to manage hyperlipidemia, hyperglycemia, and risk factors for a series of complications related to diabetes, as well as specific biomarkers.

Owing to the diversity of T2DM diseases, identifying biomarkers of T2DM has become a huge problem. They are employed to assess chemical characteristics, target validation, disease status, and the response of treatment [3]. With the progress of metabolic technology and proteomics, serum biomarkers of T2DM have existed. In addition, integrating various profiles for instance transcriptomics, proteomics, and metabolomics will better present the biological process and gene expression regulation of T2DM so as to formulate prevention strategies and reduce complications [4].

TCM is a medical system centered on medical care. It has more than 3000 years of continuous experience of practice and is improved via the treatment observation [5]. As a result, it has its own features and advantages in personalized treatment and early intervention. In the theory of TCM, syndrome differentiation (also known as pattern classification or Zheng differentiation) is the essence and basis [6]. Diagnosis is principally decided by the overall human symptoms observation, involving observation, touching, smelling, listening, and background research [7] instead of the test at the “micro” level. For the same disease, sometimes different treatment approaches are applied for the treatment of various pathological states. The relationship between these syndromes and the relevant treatment comes from practical experience and is improved through the investigations of long-term treatment. Nevertheless, due to the lack of technological and scientific means, TCM is facing serious challenges and lack of modern research [8]. Therefore, it is essential to explore the changes of compounds (containing fatty acids, proteins, metabolites, and so on) in several symptoms of a same disease in order to confirm these experiences and subsequently expand the disease understanding.

In accordance with the theory of TCM, T2DM is considered as *Xiaokezheng* having symptomatic polydipsia [8]. TCM in-depth classifies *Xiaokezheng* into distinct syndromes and has relevant clinical manifestations, containing damp-heat syndrome (DHS), *Qi Yin* deficiency, and *Qi* deficiency [9]. Based on different syndromes of TCM, TCM can provide more effective personalized treatment according to its pathological features. So far, more and more randomized clinical trials have focused on the advantages of TCM in the treatment of diabetes. In this research, we employed metabolomics-proteomics analysis to identify non-DHS and DHS syndromes of T2DM.

## 2. Materials and Methods

### 2.1. Plasma Sample Collection

The experiment was authorized through the ethics committee of the institute and complied with the principles of the Helsinki declaration. Moreover, from the patients, the informed consent to research protocol could be acquired. The plasma samples were harvested from the T2DM patients with and without the DHS syndrome (thirty in each group). In both experimental groups, all of the chose patients were diagnosed through syndrome differentiation of western medicine and TCM. They were offered via Nanjing Hospital of Traditional Chinese Medicine and adhered to the guidance of the Hospital Human Subjects Committee. The values of fasting blood glucose (FPG) were more than 7.0 mmol/L, and some blood lipid parameters, for instance, TG, TC, LDL, and HDL, were acquired via clinical detection approaches. It was classified via two authentic TCM doctors on the basis of “Diabetes TCM Diagnostic Criteria” [9].  Table 1 shows the clinical features of TCM syndromes of T2DM.

After fasting at night, venous blood was harvested as mentioned above. The buffy coat, plasma, and serum were isolated from whole blood and kept at a temperature of −80°C within four hours after collection. In order to repeat freeze-thaw cycles and maximize the longevity, serum and plasma samples were widely divided and placed at −80°C prior to the subsequent use. Levels of clinical features were determined by automated clinical laboratory methods using a diagnostic analyzer.

### 2.2. Data Processing and Metabolite Identification

The integrated and centroided data of UPLC-TOFMS were pretreated with XCMS software according to the manufacturer's recommendation [10], which was then normalized to the ionic strength of their respective internal standards in metabolomics analysis experiments [11]. The remarkably altered metabolites were identified via multivariate analysis. The Madison Metabolomics Consortium Database (MMCD) [12] as well as the Human Metabolome Database (HMDB) [13] were employed to determine the metabolites through accurate search based on mass. The identification of metabolite was demonstrated through the comparison of the retention time under identical chromatographic conditions and through the match of the cleavage mode of parent ions in biological samples with that of standard metabolites via applying tandem mass spectrometry (UPLC-TOFMS/MS).

### 2.3. TMT Proteomic Analysis

The UHPLC separation was carried out using a 1290 Infinity series UHPLC System (Agilent Technologies), equipped with a UPLC BEH Amide column (2.1 ∗ 100 mm, 1.7 *μ*m, Waters). The mobile phase consisted of 25 mmol/L of ammonium acetate and 25 mmol/L of ammonia hydroxide in water (pH = 9.75) (A) and acetonitrile (B). The analysis was carried out with elution gradient as follows: 0∼0.5 min, 95%B; 0.5∼7.0 min, 95%∼65% B; 7.0∼8.0 min, 65%∼40% B; 8.0∼9.0 min, 40%B; 9.0∼9.1 min, 40%∼95%B; and 9.1∼12.0 min, 95%B. The column temperature was25 °C. The auto-sampler temperature was 4°C, and the injection volume was 2 *μ*L (pos) or 2 *μ*L (neg), respectively. The TripleTOF 6600 mass spectrometry (AB Sciex) was used for its ability to acquire MS/MS spectra on an information-dependent basis (IDA) during an LC/MS experiment. In this mode, the acquisition software (Analyst TF 1.7, AB Sciex) continuously evaluates the full scan survey MS data as it collects and triggers the acquisition of MS/MS spectra depending on preselected criteria. In each cycle, the most intensive 12 precursor ions with intensity above 100 were chosen for MS/MS at collision energy (CE) of 30 eV. The cycle time was 0.56 s. ESI source conditions were set as following: gas 1 as 60 psi, gas 2 as 60 psi, curtain gas as 35 psi, source temperature as 600°C, declustering potential as 60 V, ion spray voltage floating (ISVF) as 5000 V or −4000 V in positive or negative modes, respectively. One QC is inserted for every 8 samples, for a total of 7 QCs.

Protein Pilot software 3.0 (ABSCIEX) was used to quantify relative abundance and identify protein and peptide. MMTS was utilized as the fixed modification of the cysteine to analyze the data, and the database could be searched with the interval rate of confidence (95%) to identify the protein. For the target proteins, their high confidence peptides revealing abundant production spectrum were chose for the assay of multiple reaction monitoring (MRM). TargetLynx 2.0 was used to treat the data of MRM, and the Graph Pad Prism program v 5.0 was employed to conduct the statistical analysis and for the generation of the receiver operating features. Each peptide was compared with the Wilcoxon test.

### 2.4. Bioinformatics Analysis

The analysis of pathway enrichment and Gene Ontology (GO) was performed for the protein functional enrichment. In accordance with the presenting report, with the InterProScan database (v.5.14–53.0 https://www.ebi.ac.uk/interpro/), GO was annotated (containing the cellular component (CC), molecular function (MF), and the biological process (BP)). The exploration of pathway enrichment was implemented with the Kyoto Encyclopedia of Genes and Genomes (KEGG) Database [14]. The KEGG mapper (v.2.5, https://www.kegg.jp/kegg/mapper.html) along with KAAS (v.2.0, https://www.genome.jp/kaas-bin/kaas_main) were the major tools employed by the database of KEGG. The WoLF PSORT software (v.0.2, https://www.genscript.com/psort/wolf_psort.html) was applied to predict the subcellular localization. The heat map obtained via the function heatmap in R language package is applied to visualize the cluster members. For each annotation, the comparison of enrichment degree between all the identified proteins and differentially abundant proteins was performed with Fisher's exact test, and *P* less than 0.05 was regarded as significant.

### 2.5. Statistical Analyses

The biochemical and clinical data were presented with mean ± SD. The SPSS program for Windows (version 21 statistical software: Texas instruments, IL, USA) was employed for all of the statistical analyses. The Mann–Whitney/Wilcoxon and Student's *t*-test were exploited to carry out the differences between both TCM syndrome groups of T2DM when proper. The two-tailed *p* value is significant when FDR-adjusted*p* < 0.05.

## 3. Results

### 3.1. Clinical Data of the Study Subjects

The biochemical and clinical data of the study subjects are reflected in Table 2. The DHS group had significantly higher values for TG (*P* < 0.001), TC (*P* < 0.05), fasting insulin (FINS, *P* < 0.05), fasting c-peptide (FCP, *P* < 0.05), and insulin resistance index (HOMA-IR, *P* < 0.05). Furthermore, the DHS group showed a lower mean level of HDL and a higher level of LDL than the non-DHS group, although there was no statistical difference.

### 3.2. Identification and Functional Enrichment Analysis of Differentially Abundant Proteins

**Figure 1:**
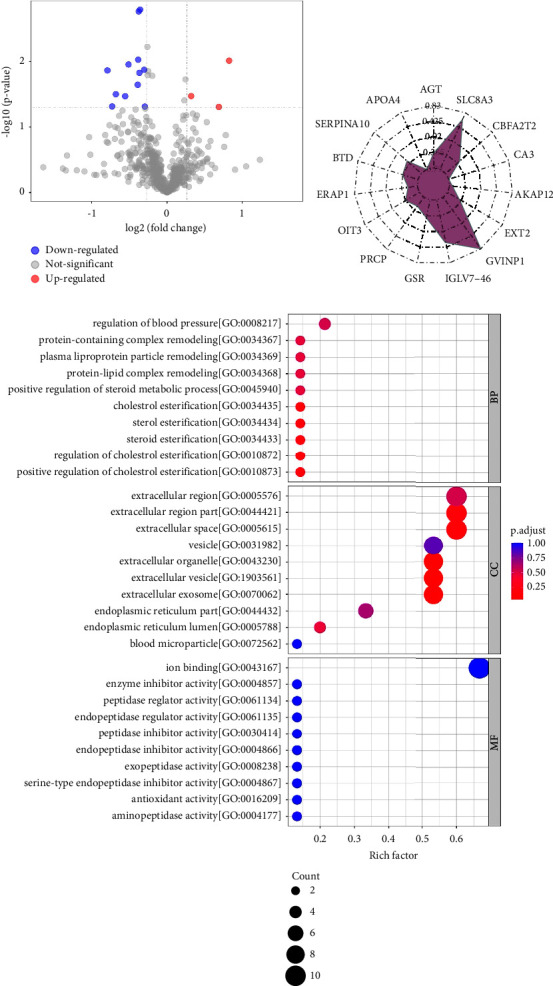
The analysis of functional enrichment of the differentially abundant proteins in T2DM patients with/without damp-heat syndrome (DHS). (a) The volcano map exhibited differentially abundant proteins (DHS vs. non-DHS group). Remarkably differentially abundant proteins were coded by color: red and blue represent increased and decreased proteins, respectively. Two clusters composed of twelve decreased and three increased proteins. (b) Radar chart analysis for group DHS vs. non-DHS. The conversion value of log2 was utilized as a baseline, and the corresponding quantitative value ratio of differential protein was calculated. The black number in the figure indicated the fold change. (c) The bubble chart reflected the classification outcomes of the top 15 most remarkable enrichment, containing molecular function, the cellular component, and the biological process (*P* < 0.05). In bubble chart, the vertical axis represented the pathway or functional classification, and the horizontal axis represented the enrichment factor, which represented the comparison between the proportional value of identified proteins and the proportional value of differentially rich proteins in the functional type. The color of circle showed the enrichment *P* value, and the size of circle reflected the differentially abundant protein number in pathways or functional categories.

Using the abovementioned analytical conditions, proteomic profiles from 30 T2DM patients with TCM syndrome with DHS and 30 without DHS were obtained by LC-MS/MS. There were 654 proteins available. After data management and normalization, there were 621 quantifiable proteins (Figure 1). In the end, fifteen proteins revealed obvious differences (*P* < 0.05; FCa0< 0.83 or >1.2), of which 3 proteins were increased and 12 proteins were decreased (Figures 1(a) and 1(b)). With the aim of determining the features of differentially abundant proteins, the analysis of KEGG and GO of proteins were annotated.

BP classification of the proteins suggested that various abnormal biological processes appeared in the syndrome of DHS, for instance positive regulation or regulation of the cholesterol esterification, sterol or steroid esterification, positive regulation of the steroid metabolism, and protein-lipid complex remodeling (Figure 1(c)). Remarkably, representative differentially abundant proteins including AGT and APOA4 participated in all the above process.

The classification of protein via CC indicated that most differentially expressed proteins are located in the extracellular regions, even in the extracellular organelles or vesicles. The classification of protein via MF indicated that more proteins were associated with iron binding, followed by cation binding, aminopeptidase activity, antioxidant activity, exopeptidase activity, and so on (Figure 1(c)). The following analysis of KEGG suggested that the most remarkable change pathways involved the renin-angiotensin system, vitamin digestion and absorption, hypertrophic cardiomyopathy, dilated cardiomyopathy, protein digestion and absorption, adrenergic signaling in cardiomyocytes, and metabolic pathways. In conclusion, extensive and comprehensive proteomic data revealed that many metabolic pathways of T2DM were abnormal in the syndrome of DHS.

### 3.3. Changes in Serum Metabolites Detected by Untargeted Metabolomics

**Figure 2:**
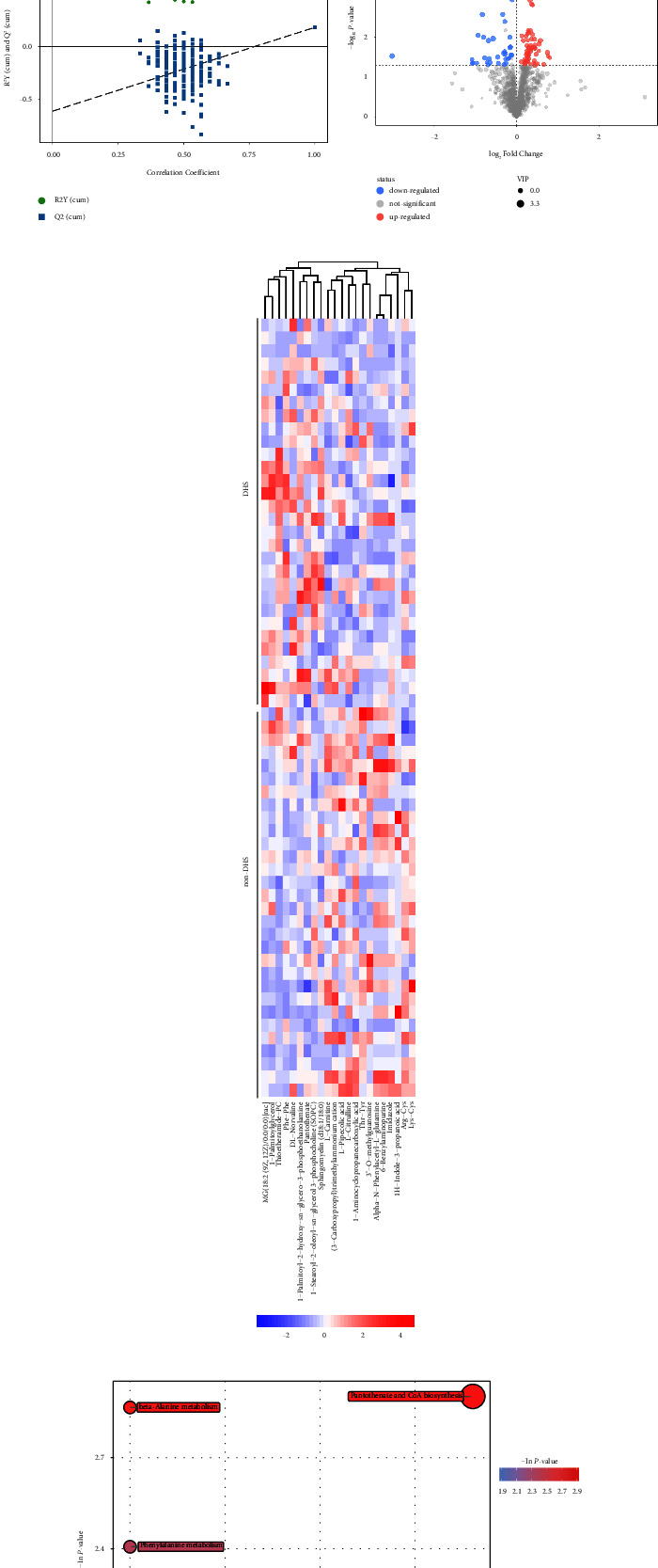
Metabolomics analysis of serum samples in T2DM patients with / without damp-heat syndrome (DHS). (a) Two-dimensional PCA score plot representation for QC samples, red dots represented non-DHS samples and blue dots represented DHS samples. The position of the point in the graph was determined by all the metabolites in the sample. (b) Score scatter plot of OPLS-DA model for group DHS (red) vs. non-DHS (blue). (c) Permutation test of OPLS-DA model for group DHS vs. non-DHS. The metabolomics analysis of serum samples from T2DM patients with or without DHS. In the figure, the horizontal axis represented the retention of the replacement test, the longitudinal axis represented the value of R2Y or Q2, the green dot represented the R2Y value acquired from replacement detection, the blue square point was the value of Q2. (d) The volcano diagram exhibited differentially abundant metabolites (DHS vs. Non-DHS group). Remarkably differentially abundant metabolites were encoded via color: red and blue were represented upregulated and downregulated metabolites, respectively. Two clusters composed of twenty-two metabolites revealing significant differences. (e) The clustering heat plot for differentially abundant metabolites. The vertical and horizontal axis respectively reflected differentially abundant metabolites and sample. Red and blue represented positive and negative correlation, respectively. (f) The analysis of enrichment of differentially abundant pathways. The pathway name was displayed; the higher the red intensity, the smaller the value of P; the larger size of circle indicated higher impact.

Relative standard deviation denoising was conducted (individual peaks were filtered to remove noise, and the deviation value was filtered based on the relative standard deviation). 1895 peaks were determined. In addition, the approach of internal standard normalization was employed in analyzing the data. The data were scaled and then transformed logarithmically to minimize the influence of variable high variance and noise. After above transformations, the grouping and distribution of samples was visualized with principal component analysis (PCA). The confidence interval (95%) in the PCA score graph was employed as a threshold to determine latent outliers in the data set. The outcomes indicated that our present metabolomics data set has outstanding reproducibility and stability (Figure 2(a)). Orthogonal projection with latent structures discriminate analysis (OPLS-DA) was performed to choose remarkably changed metabolites between non-DHS and DHS groups (Figure 2(b)). Subsequently, the calculation of the *Q*^2^ and *R*^2^ values was carried out with a 7-fold cross-validation, respectively, suggesting how well the variables were predicted and the changes in variables were explained. In order to test the prediction ability and robustness of the OPLS-DA model, 200 permutations were carried out in-depth, and subsequently, the intercept values of *Q*^2^ and *R*^2^ were acquired. Here, the *Q*^2^ intercept value was the model reliability, the risk of over fitting, and the model robustness; the smaller the better (Figure 2(c)). In addition, the first principal component, the variable importance in the projection (VIP) value in the analysis of OPLS-DA, was acquired (Figure 2(d)). Metabolites with *P* < 0.05, FC > 1, and VIP >1 (Student's *t*-test) were regarded to be remarkably changed metabolites. In the end, 22 metabolites which revealed significant differences in DHS (FC > 1.2 and *P* < 0.05) were determined. As shown in Figure 2(e), imidazole, L-pipecolic acid, L-citrulline, L-carnitine, and 3′-O-methylguanosine were decreased, while pantothenate, sphingomyelin, and thioetheramide-PC were increased. Furthermore, the subsequent metabolic pathways had significantly changed: the biosynthesis of CoA and pantothenate, the metabolisms of phenylalanine, beta-alanine, proline, and arginine (Figure 2(f)). These outcomes exhibited that vitamin and amino acid metabolism were changed in T2DM patients with the syndrome of DHS. These analyses were performed by using SIMCA (16.0.2) software.

### 3.4. Combination Analysis of Proteomics and Metabolomics Data

**Figure 3:**
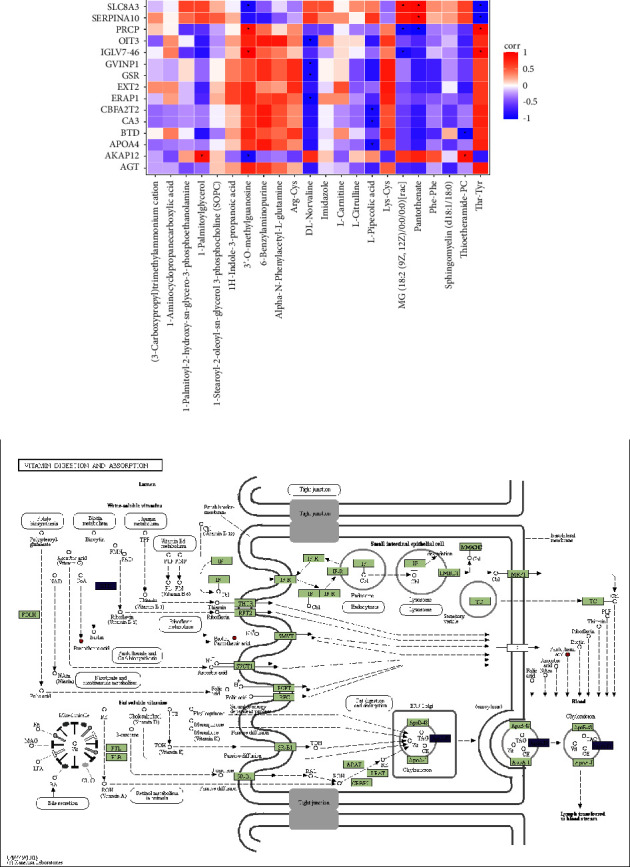
The combination of metabolomics and proteomics data. (a) The clustering heat map of relative results. The vertical axis and horizontal axis, respectively, were the differentially abundant proteins and differentially abundant metabolites. Red and blue represented positive and negative correlation, respectively. ^*∗*^*P* < 0.05. (b) Visual analysis of KEGG maps about vitamin digestion and absorption.

The outcomes of LC-MS/MS were analyzed with Paintomics3 (v.0.4.5, http://www.paintomics.org). We calculated the content information of all differentially expressed proteins and metabolites, compared the correlation between them, and used Spearman rank and rank correlation to analyze the different metabolites and proteins. We took the correlation coefficient *Q*-value <0.05 as the condition of significant correlation. The heatmap of proteomic-metabolomic correlation analysis for group DHS vs. non-DHS revealed that SLC8A3 and SERPINA10 had a positive relationship with pantothenate and negative with Thr-Tyr, and lysosomal pro-X carboxypeptidase (PRCP) had positive correlation with 3′-O-methylguanosine and Thr-Tyr and negative with pantothenate. DL-Norvaline was negatively related to oncoprotein-induced transcript 3 (OIT3), interferon-induced very large GTPase 1 (GVINP1), glutathione reductase (GSR), and endoplasmic reticulum aminopeptidase 1 (ERAP1). L-Pipecolic acid was negatively related to CBFA2T2, carbonic anhydrase 3 (CA3), and APOA4. Thioetheramide-PC was positively related with A-kinase anchor protein 12 (AKAP12) and negatively with biotinidase (BTD) (Figure 3(a)). Through the visual analysis of the KEGG diagram, it can be concluded that metabolites and protein were jointly regulated. The vitamin digestion and absorption pathway exhibited downregulation of APOA4 (*P* < 0.05, FC = 0.60), BTD (*P* < 0.05, FC = 0.76) and upregulation of pantothenate (*P* < 0.05, FC = 1.20) (Figure 3(b)). The integrated analysis outcomes indicated the potential mechanism of vitamin metabolism in T2DM patients with DHS syndrome.

## 4. Discussion

Omics are generally employed to elucidate the diabetes pathogenesis and screen latent phenotypic markers of T2DM. In comparison with single omics, integrated analysis of multiomics (the integrated analysis of metabolomics and proteomics [15] or metabolomics and transcriptomics [16], etc.) can more deeply reveal the molecular characteristics and physiological mechanism of this disease. In our work, DHS produced a lot of data on the proteomic analysis of T2DM patients' serum, reflecting the mechanisms of diabetes occurrence and development, for example the renin-angiotensin system, vitamin digestion and absorption, hypertrophic cardiomyopathy, dilated cardiomyopathy, protein digestion and absorption, adrenergic signaling in cardiomyocytes, and metabolic pathways. Serum metabolites can exhibit the body metabolic changes, which is helpful to the monitor of metabolic process in the state of disease [17]. With the aim of understanding the whole metabolic status of T2DM patients with DHS, we implemented a metabolomic analysis on the serum samples and acquired the metabolic profiles. Besides, we implemented an integrated metabolomic and proteomic analysis.

Long-term exposure to high blood glucose could enhance the risk of amputation, heart attacks, diabetic retinopathy as well as strokes. Intuitively, T2DM could be classified into several cases without or with the complications. Nevertheless, so far, T2DM has not been classified in accordance with the clinical parameters. Interestingly, various syndromes of T2DM can be decided via the theory of TCM, despite they are on the basis of long-term experience of practical. More significantly, special treatment is generally carried out under the guidance of various syndrome types. As a result, with the aim of further understanding T2DM, it is essential to prove various syndromes through biological approaches, especially the analysis of omics with big data. In this study, two syndromes were explored applying the method of metabolomics-proteomics analysis, together with the clinical data including glucose measurements and four lipid parameters (TG, TC, and HDL together with LDL). PCA and OPLS-DA were carried out to, respectively, construct the discriminant models for the T2DM patients with two syndromes of TCM and next explore the correlation between syndromes and proteomics-metabolites. OPLS-DA is a complex and generally applied supervised clustering approach, which is employed to construct the best discriminant surface in order to isolate the best classification. The difference between the two groups was significant, and with Hotelling's *T*-square ellipse, the samples were basically within the confidence interval of 95%. Thus, the statistical analysis results indicated that the plasma metabolic profiles could exhibit some perturbations between DHS and non-DHS syndromes in TCM.

Many latent biomarkers of various syndromes have been found, suggesting that distinct disease pathological stages may be associated with the metabolic status closely. For both groups, DHS, vs. non-DHS, AGT, APOA4, SERPINA10, BTD, ERAP1, OIT3, PRCP, GSR, IGLV7-46, GVINP1, EXT2, AKAP12, CA3, and CBFA2T2 (*P* < 0.05; FC < 0.83 or >1.2) were the potential biomarkers. 6-Benzylaminopurine, alpha-N-phenylacetyl-L-glutamine, pantothenate, thioetheramide-PC, 1-stearoyl-2-oleoyl-sn-glycerol3-phosphocholine (SOPC), 1-palmitoylglycerol, 1H-indole-3-propanoic acid, imidazole, L-pipecolic acid, 3′-O-methylguanosine (*P* < 0.05; FC < 0.83 or >1.2, VIP >1.3) were the candidate metabolic biomarkers. These biomarkers are the major characteristics of this syndrome. The changes of their content are linked to the human symptoms of T2DM and can be employed to distinguish a variety of syndromes. According to Table 2, it is understandable why DHS patients with lower APOA4 exhibit significantly elevated TC and TG. The upregulated FCP, FINS, and HOMA-IR also reflect the worsening process of T2DM. It is an effective and innovative method to divide the disease into distinct stages in accordance with a variety of symptoms and then carry out the symptom-specific treatment. In addition, the observation of syndrome-related biomarkers is especially significant for the personalized treatment. Biomarkers related to syndrome of TCM can assist the clinical diagnosis, promote the modernization of TCM, and offer a reference for exhibiting the diabetes pathogenesis.

The proteomics analysis revealed that differentially abundant proteins were related to the vitamin digestion and absorption. After integrated study of the metabolomics and proteomics outcomes, it can be observed that both proteins (APOA4 and BTD) in the DHS group were downregulated as well as one amino acid metabolite (pantothenate) was upregulated in the serum, thus affecting the vitamin metabolism pathway. It has been reported that the levels of plasma vitamin C in patients with T2DM were relatively low, which made people pay growing attention to the preventive effect of vitamin C on T2DM and its related complications. The prospective cohort survey in seven countries exhibited that the glucose intolerance was negatively correlated with the intake of dietary vitamin C, reflecting that antioxidants, for example vitamin C, may possess a protective effect in the occurrence of T2DM and impaired glucose tolerance [18]. In addition, some clinical researches have confirmed that the supplementation of vitamin D can improve the principal metabolic parameters linked to the insulin resistance, containing HbA1c, TC, LDL, triglycerides, as well as HOMA-IR. The supplementation of vitamin D for three months in the elderly with the metabolic disorders can evidently increase the level of HDL and decrease the ratio of TG/HDL and HOMA-IR [19], which is also supported by our results. Similarly, the percentage of HbA1c in T2DM patients decreased by approximately 0.5% after the supplementation of vitamin D [20]. Pantetheine, also called vitamin B5, plays a crucial role in the CoA biosynthetic pathway. It presents good opportunity for drug discovery moving forward [21]. Since B vitamins are the cornerstone of all the cell repair, during the first two years of the supplementation of vitamin D, the increase of repair and the improvement of sleep ultimately leaded to the consumption of B5 reserves. Owing to the lack of B5, the generation of coenzyme A in the brain was reduced, which may lead to the reduction of acetylcholine generation, resulting in sleep disorders. The decrease in CoA in the adrenal gland may lead to the reduction of the cortisol level (adrenal fatigue), resulting in the increase in arthritis, allergy, and inflammation [22]. As the DHS group showed lower APOA4 and higher pantothenic, it is understandable that non-DHS patients feature sleep hyperhidrosis, fatigue, as well as joint disorders. In the light of lots of articles [23–25] connecting vitamin D with the normal immune system function and the fact that B5 is essential for cortisol production, it can be speculated that the continuous deficiency of B5 and vitamin D may lead to the proinflammatory and abnormal state in patients with T2DM.

As the metabolomics was “untargeted” and the number of significant features in both the proteins and the metabolomics was not very many, further work and large follow-up studies are required to remedy the defect of this research.

## 5. Conclusion

To sum up, via implementing metabolomics and proteomics analysis of the serum of T2DM patients with or without DHS, we gave an initial understanding of the features of diabetes with two different TCM syndromes from a molecular perspective. Furthermore, we found that, in the DHS patients, the metabolic abnormalities were especially prominent, particularly vitamins metabolism. These results provided a fundamental data for the extensive application of TCM in the study of T2DM and at the same time benefited in a sense diagnosis and treatment of T2DM.

## Figures and Tables

**Table 1:** The clinical features of TCM syndromes of T2DM.

	*Qi* deficiency	*Qi*and *Yin* deficiency	Damp heat
Representative symptoms	Lethargy	Soreness of waist and knees	Thirsty
	Constipation	Sleep hyperhidrosis	Diuresis

Holistic symptoms	Fatigue	Palpitations and insomnia	Hyperorexia

Tongue appearance	Fat tongue	Reddish tongue	Red tongue with yellow fur

Pulse pattern	Thin and weak pulse	Thin and rapid pulse	Stringy pulse

**Table 2:** Clinical and biochemical data of the study subjects.

Characteristics	T2DM with non-DHS (*n* = 30)	T2DM with DHS (*n* = 30)	*p* value
Age (years)	57.80 ± 11.82	52.97 ± 11.22	0.11
BMI (kg/m^2^)	25.92 ± 3.28	26.70 ± 3.65	0.39
FBG (mmol/L)	9.48 ± 3.11	9.82 ± 2.68	0.65
PBG (mmol/L)	14.50 ± 4.12	15.00 ± 4.11	0.64
HbA1c (%)	9.16 ± 1.70	9.42 ± 1.59	0.54
TC (mM)	4.38 ± 1.28	5.57 ± 1.41	≤0.01
TG (mM)	1.58 ± 1.10	3.10 ± 1.79	≤0.01
LDL-C (mM)	2.79 ± 0.83	3.14 ± 1.14	0.18
HDL-C (mM)	1.13 ± 0.22	1.00 ± 0.29	0.06
FCP (pg/mL)	2.02 ± 0.67	2.62 ± 1.11	≤0.01
FINS (pmol/ml)	8.15 ± 5.08	12.24 ± 6.93	≤0.01
HOMA-IR	3.61 ± 3.10	5.44 ± 3.27	0.03

Data were presented as means ± SD, and *t*-test was applied. The two-tailed *p* value was significant at <0.05. DHS: damp-heat syndrome; BMI: body mass index; FBG: fasting blood glucose; PBG: postprandial plasma glucose; HbA1c: glycosylated hemoglobin; TC: total cholesterol; TG: triglycerides; LDL-C: low-density lipoprotein cholesterol; HDL-C: high-density lipoprotein cholesterol; FCP: fasting c-peptide; FINS: fasting insulin; HOMA-IR: insulin resistance index.

## Data Availability

The quality control and omics data used to support the findings of this study are available from the corresponding author upon request.
